# Virtual Horizons in Medicine: The Role of the Metaverse in Transforming Medical Education—A Qualitative Scoping Review

**DOI:** 10.1002/hsr2.73267

**Published:** 2026-09-23

**Authors:** Fariba Khanipoor, Zahra Karimian

**Affiliations:** ^1^ Student Research Committee Shiraz University of Medical Sciences Shiraz Iran; ^2^ Department of E‐Learning in Medical Sciences, Virtual School and Center of Excellence in E‐Learning Shiraz University of Medical Sciences Shiraz Iran

**Keywords:** Arksey and O'Malley Protocol, CASP tool, medical education, metaverse, qualitative approach, thematic analysis

## Abstract

**Background:**

The metaverse is defined as a three‐dimensional, interactive and persistent virtual space in which users interact with one another and with simulated environments through digital avatars. This technology, as an innovative approach to education and learning, holds transformative potential for medical education. This qualitative scoping review aimed to identify, analyze, and synthesize qualitative evidence on stakeholders' perspectives and experiences regarding the application, role and significance of the metaverse in medical education.

**Methods:**

This scoping review was conducted following the six‐stage framework proposed by Arksey and O'Malley. A comprehensive and systematic search was performed across English‐ and Persian‐language databases to identify evidence related to the application of the metaverse in medical education. English‐language databases included Web of Science, PubMed, Scopus, ERIC, ACM Digital Library, and IEEE Xplore. To encompass studies published in Persian, databases such as SID (Scientific Information Database), Civilica, Magiran, and ElmNet were searched. Additionally, Google Scholar was utilized to ensure the comprehensiveness of the search. English keywords included Metaverse, Medical Education, Qualitative, and related combinations. For standardized searches in databases like PubMed, keywords were aligned with Medical Subject Headings (MeSH*)* terms. The temporal scope of the studies ranged from January 1, 2015, to December 30, 2024. The PRISMA‐ScR framework was employed to document the process of study identification and screening. Methodological quality was assessed using the Critical Appraisal Skills Programme tool (CASP 2018).

**Results:**

Ultimately, twelve studies met the inclusion criteria. The findings revealed that the majority of articles were published in 2024, with a significant proportion originating from Asia. Eight key themes related to the metaverse in medical education were identified: immersion and sense of reality in the metaverse, educational benefits of the metaverse, technical and design challenges, privacy and security concerns, social and psychological impacts, accessibility and inclusivity, regulatory and ethical frameworks, and educational innovations and teaching methodologies.

**Conclusion:**

The evidence from this qualitative review of stakeholders indicates that the metaverse is currently valued in medical education primarily as an immersive complement to conventional instruction. Technical challenges, privacy concerns, access issues, and the need for robust regulatory frameworks must be addressed. Without these, technological expansion may widen inequality and erode trust. Most available evidence is confined to short‐term learner experience, and little is yet known about how the technology alters durable learning, long‐term mental health, or the implementation of educational policy.

**Abbreviations**
SID: Scientific Information Database
MeSH: Medical Subject Headings
PRISMA‐ScR: PRISMA for Scoping Reviews
CASP: Critical Appraisal Skills Program
AR: Augmented Reality
VR: Virtual Reality

## 1 Introduction

Medical education is continuously evolving and advancing [1]. Education in the health sciences plays a critical role and must be prioritized across all levels of service delivery [2]. Given its complex nature and the necessity for practical skills and clinical interactions, medical education consistently requires innovative approaches to enhance learning quality and expand access to educational resources [3]. Traditional constraints in medical education, such as the high costs of physical simulators, limited access to real patients, and geographical barriers, have underscored the need for advanced technological solutions [4]. Over the past decade, digital technological advancements have profoundly transformed medical education [5]. Among these, the metaverse, as an emerging technology, has introduced unprecedented opportunities to redefine teaching and learning methodologies in the health sciences. The metaverse is defined as a three‐dimensional, interactive, and persistent virtual space where users, through digital avatars, engage with one another and with simulated environments [6]. It represents a parallel digital ecosystem in which activities such as education, collaboration, and complex simulations occur simultaneously on a global scale [7].

Among innovative learning methodologies in medical education, the metaverse occupies a distinctive position. Often described as a revolutionary development in medical education, the metaverse is rapidly expanding and is anticipated to dominate future educational landscapes [8]. As previously noted, the metaverse is a digital realm where users interact via virtual avatars [9]. Within this environment, individuals can perform actions that may be infeasible in the physical world, such as simulating complex surgical procedures or engaging in interactive educational experiences [10, 11]. The metaverse enhances communication applications [12], social interactions [13], educational and learning processes [14, 15, 16, 17], professional performance, and comprehension in the medical domain [10, 11, 18]. Furthermore, by providing advanced simulated environments, the metaverse enables the training of clinical skills, the execution of virtual surgeries, and the fostering of interdisciplinary collaboration. For instance, research has demonstrated that the metaverse can facilitate virtual operating rooms, allowing medical students to practice surgical techniques in a safe and controlled setting [19, 20]. The COVID‐19 pandemic further highlighted the metaverse's role in medical education. Restrictions imposed by social distancing and the demand for remote education transformed digital technologies into an essential component of training [21]. As a platform for interactive and remote education, the metaverse has enabled functionalities such as online medical classes, three‐dimensional anatomical simulations, and virtual consultation sessions [22].

### 1.1 Research Aim and Question

Despite these advancements, the scientific literature on the role and importance of the metaverse in medical education remains in its early stages. Recent and systematic reviews have consistently shown that the majority of existing studies primarily focus on technical feasibility, quantitative outcome measures, or limited applications such as surgical simulation, while qualitative evidence examining the experiences and perspectives of key stakeholders remains scarce [23, 24, 25]. Specifically, only a small portion of published works (approximately 9% in comprehensive reviews of more than 170 studies) employ qualitative methods to capture how educators, students, and healthcare professionals perceive, experience, and navigate the metaverse in educational contexts [24]. The scarcity of qualitative studies that integrate the perspectives of stakeholders (students, instructors, and healthcare professionals) has created a significant research gap. Without a synthesized understanding of stakeholder perspectives, the pathway from educational innovation to measurable clinical impact has not been well mapped.

Scoping reviews are recognized as an appropriate research methodology for exploring emerging and complex fields, as they enable the identification of knowledge gaps, the examination of a topic's scope, and the provision of a comprehensive overview of existing research (Arksey & O'Malley, 2005) [26]. The aim of this qualitative scoping review is to identify, analyze, and synthesize findings from original qualitative articles and the qualitative components of mixed‐methods studies on stakeholders' perspectives and experiences—including educators, students, and healthcare professionals— concerning the application. role and significance of the metaverse in medical education. Given the novelty of this technology and the diversity of its applications, integrating these perspectives appears essential. Accordingly, the primary research question guiding this study is: What is the application, role and significance of the metaverse in medical education from the perspectives and experiences of stakeholders?

## 2 Methods

This study was designed as a qualitative scoping review to examine and synthesize qualitative evidence on stakeholders' perspectives and experiences related to the application, role and significance of the metaverse in medical education. The review adheres to the six‐stage framework outlined by Arksey and O'Malley (2005), encompassing the following steps: (a) identifying the research question, (b) identifying relevant studies, (c) selecting studies, (d) charting the data, (e) collating, analyzing, and reporting results, and (f) consulting with stakeholders (optional).

### 2.1 Stage 1: Identifying the Research Question

The objective of this study is to provide a comprehensive overview of the current application, role and status of the metaverse in medical education of stakeholders' perspectives and experiences globally. The primary research question guiding this review is: “What is the application, role and significance of the metaverse in medical education from the perspectives and experiences of stakeholders?”

### 2.2 Stage 2: Identifying Relevant Studies

To identify qualitative evidence pertinent to the application, role and significance of the metaverse in medical education, a systematic and comprehensive search was conducted across English‐ and Persian‐language databases. English‐language databases included Web of Science, PubMed, Scopus, ERIC, ACM Digital Library, and IEEE Xplore. To ensure coverage of studies published in Persian, databases such as SID (Scientific Information Database), Civilica, Magiran, and ElmNet were searched. Additionally, Google Scholar (Search engine) was utilized to enhance the thoroughness of the search. English keywords included Metaverse, Medical Education, Qualitative, and related combinations. For standardized searches in databases such as PubMed, keywords were aligned with Medical Subject Headings (MeSH) terms, as detailed below:

*Entry Terms for Metaverse:* Metaverse, Virtual Reality (VR), Augmented Reality (AR), Cyberspace, Multiverse, Virtual Worlds, Computer Simulation, Information Technology

*Entry Terms for Medical Education:* Medical Education, (Education, Undergraduate Medical), (Medical Education, Undergraduate), Undergraduate Medical Education, Graduate Medical Education, (Medical Education, Graduate), (Education, Graduate Medical), Continuing Medical Education, (Education, Continuing Medical), (Medical Education, Continuing)

**Figure 1:**
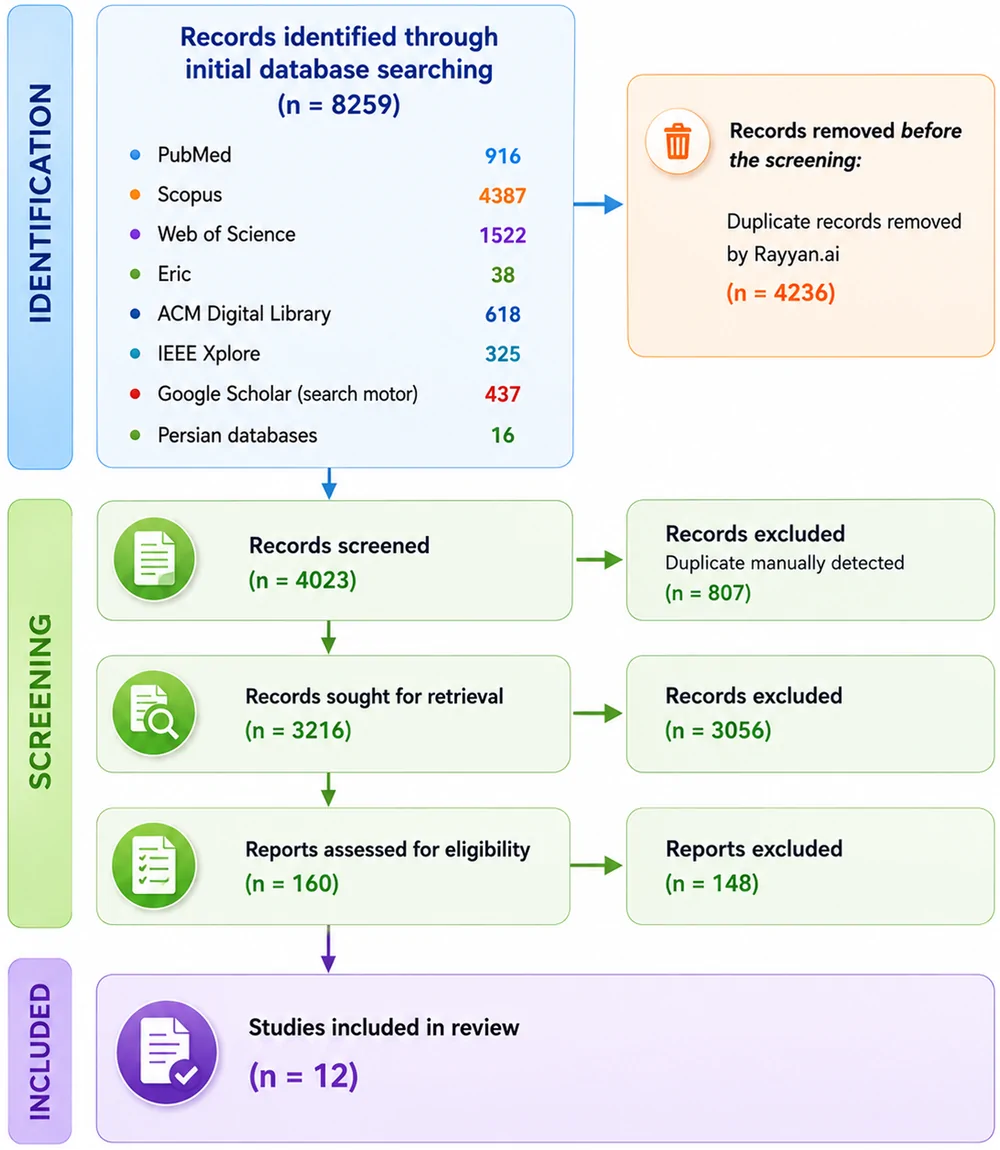
PRISMA‐ScR flow diagram of the study selection process.

Persian equivalents of these keywords included Metaverse, Medical Education, Health Sciences Education, Virtual Reality, and Augmented Reality in Medical Education. Search strategies were tailored for each database (Table 1). To optimize precision and coverage, Boolean operators (AND, OR, NOT) were employed. For instance, in Persian‐language databases, the search string was structured as: (Metaverse *OR* Virtual Reality) AND (Medical Education *OR* Health Sciences Education). The temporal scope of the search spanned from January 2015 to December 2024. The selection of 2015 as the starting point is justified by significant advancements in virtual reality (VR) and augmented reality (AR) technologies, such as the introduction of Oculus Rift and Microsoft HoloLens, which enabled the development of metaverse environments [27]. This timeframe also aligns with societal shifts, such as the digitization of education and the emergence of the Education 4.0 movement [28]. Thus, the 2015–2024 period facilitates a comprehensive examination of the emerging literature and the identification of knowledge gaps in this domain. The PRISMA‐ScR (Preferred Reporting Items for Systematic Reviews and Meta‐Analyses for Scoping Reviews) framework was used to document the study identification and screening process. A PRISMA‐ScR flow diagram was employed to illustrate the various stages of the search, from initial identification to final inclusion of studies (Figure 1).

**Table 1:** Search terms utilized in Academic Databases.

Database	Search term used
PubMed	(“Metaverse”[Title/Abstract] OR “Virtual Reality”[Title/Abstract] OR “Augmented Reality”[Title/Abstract] OR “Cyberspace”[Title/Abstract] OR “Multiverse”[Title/Abstract] OR “Virtual Worlds”[Title/Abstract] OR “Computer Simulation”[Title/Abstract] OR “Information Technology”[Title/Abstract]) AND (“Medical Education”[Title/Abstract] OR “Education, Undergraduate Medical”[Title/Abstract] OR “Medical Education, Undergraduate”[Title/Abstract] OR “Undergraduate Medical Education”[Title/Abstract] OR “Graduate Medical Education”[Title/Abstract] OR “Medical Education, Graduate”[Title/Abstract] OR “Education, Graduate Medical”[Title/Abstract] OR “Continuing Medical Education”[Title/Abstract] OR “Education, Continuing Medical”[Title/Abstract] OR “Medical Education, Continuing”[Title/Abstract]) AND (“2015/01/01”[Date ‐ Publication]: “2024/12/31”[Date ‐ Publication])
Scopus	(TITLE‐ABS‐KEY(“Metaverse” OR “Virtual Reality” OR “Augmented Reality” OR “Cyberspace” OR “Multiverse” OR “Virtual Worlds” OR “Computer Simulation” OR “Information Technology”)) AND (TITLE‐ABS‐KEY(“Medical Education” OR “Undergraduate Medical Education” OR “Medical Education, Undergraduate” OR “Undergraduate Medical Education” OR “Graduate Medical Education” OR “Medical Education, Graduate” OR “Education, Graduate Medical” OR “Continuing Medical Education” OR “Education, Continuing Medical” OR “Medical Education, Continuing”)) AND PUBYEAR > 2014 AND PUBYEAR < 2025
Web of sciences	TS = (“Metaverse” OR “Virtual Reality” OR “Augmented Reality” OR “Cyberspace” OR “Multiverse” OR “Virtual Worlds” OR “Computer Simulation” OR “Information Technology”) AND TS = (“Medical Education” OR “Undergraduate Medical Education” OR “Medical Education, Undergraduate” OR “Undergraduate Medical Education” OR “Graduate Medical Education” OR “Medical Education, Graduate” OR “Education, Graduate Medical” OR “Continuing Medical Education” OR “Education, Continuing Medical” OR “Medical Education, Continuing”) AND PY = (2015–2024)
Eric	(“Metaverse” OR “Virtual Reality” OR “Augmented Reality” OR “Cyberspace” OR “Multiverse” OR “Virtual Worlds” OR “Computer Simulation” OR “Information Technology”) AND (“Medical Education” OR “Undergraduate Medical Education” OR “Medical Education, Undergraduate” OR “Undergraduate Medical Education” OR “Graduate Medical Education” OR “Medical Education, Graduate” OR “Education, Graduate Medical” OR “Continuing Medical Education” OR “Education, Continuing Medical” OR “Medical Education, Continuing”)
ACM digital library	(“Metaverse” OR “Virtual Reality” OR “Augmented Reality” OR “Cyberspace” OR “Multiverse” OR “Virtual Worlds” OR “Computer Simulation” OR “Information Technology”) AND (“Medical Education” OR “Undergraduate Medical Education” OR “Medical Education, Undergraduate” OR “Undergraduate Medical Education” OR “Graduate Medical Education” OR “Medical Education, Graduate” OR “Education, Graduate Medical” OR “Continuing Medical Education” OR “Education, Continuing Medical” OR “Medical Education, Continuing”)
IEEE Xplore	(“Metaverse” OR “Virtual Reality” OR “Augmented Reality” OR “Cyberspace” OR “Multiverse” OR “Virtual Worlds” OR “Computer Simulation” OR “Information Technology”) AND (“Medical Education” OR “Undergraduate Medical Education” OR “Medical Education, Undergraduate” OR “Undergraduate Medical Education” OR “Graduate Medical Education” OR “Medical Education, Graduate” OR “Education, Graduate Medical” OR “Continuing Medical Education” OR “Education, Continuing Medical” OR “Medical Education, Continuing”)

*Inclusion Criteria**:***

- Articles published between January 2015 and December 2024.
- Articles published in English or Persian.
- Studies containing keywords related to the metaverse and medical education in their title, abstract, or keywords.
- Studies directly or indirectly addressing the research question: “What is the role and significance of the metaverse in medical education?”

*Exclusion Criteria:*

- Articles employing non‐qualitative or non‐mixed‐methods methodologies (e.g., quantitative studies, narrative reviews).
- Qualitative or mixed‐methods articles for which full texts were unavailable.
- Qualitative or mixed‐methods studies (specifically their qualitative components) that did not explicitly address the application of the metaverse in medical education (e.g., metaverse in non‐medical education or non‐educational contexts).

- Non‐article sources, such as books, book chapters, or dissertations.
- Qualitative or mixed‐methods articles published in languages other than English or Persian.

These criteria were designed to focus on relevant and credible scientific evidence. The search and screening processes were independently conducted by two researchers to ensure accuracy and minimize bias in study selection.

### 2.3 Stage 3: Study Selection

In this stage, the study selection process followed a structured, multi‐stage approach in accordance with the PRISMA‐ScR guidelines to ensure transparency and reproducibility. This process proceeded sequentially from initial identification to final inclusion as follows:

*Initial identification:* All records retrieved from systematic searches in English‐ and Persian‐language databases (Web of Science, PubMed, Scopus, ERIC, ACM Digital Library, IEEE Xplore, SID, Civilica, Magiran, ElmNet) and the Google Scholar search engine that employed relevant keywords or synonymous terms were identified and imported into EndNote software. Duplicate records were removed.

*Title screening:* Two independent researchers screened the titles of all unique records against the predefined inclusion and exclusion criteria. For example, studies that clearly did not address the metaverse in medical education or that were not qualitative/mixed methods were excluded at this stage.

*Abstract screening:* The abstracts of the remaining records were independently reviewed by the same two researchers. Articles that did not meet the eligibility criteria (e.g., non‐qualitative methodology, lack of focus on medical education) were excluded from the study.

*Full‐text screening and eligibility assessment:* The full texts of potentially eligible articles were obtained and independently evaluated by two researchers to ensure accuracy and minimize bias. Each study was assessed with respect to qualitative methodology (or the qualitative component of mixed‐methods designs), relevance to the research question, and reporting quality. To ensure the validity of the studies, only articles published in reputable journals were selected, which were obtained directly from the journals' websites or from the full‐text links provided by the databases. Articles that had been retracted or published in suspicious outlets (e.g., predatory journals) were excluded. In this study, predatory journals were defined and identified according to standard international criteria (including recognized lists such as Beall's list and the COPE and DOAJ checklists). That is, journals that lacked a transparent and valid peer‐review process, were not indexed in reputable scientific databases, charged high publication fees without quality assurance, and often had a history of publishing low‐quality or rejected articles. Articles published in such journals or rejected articles were completely excluded from the selection process so that only valid, high‐quality evidence would enter the analysis. The methodological quality of the studies was assessed using the Critical Appraisal Skills Programme (CASP) tool to confirm that the selected studies possessed robust designs and reliable findings (CASP, 2018) [29].

*Resolution of disagreements and final selection:* In the event of any disagreement, a third reviewer (an experienced faculty member in the field of medical education) was designated for the final decision. However, no disagreement was observed between the two primary reviewers in this study. Inter‐rater agreement was calculated and reported using Cohen's kappa coefficient of 0.836 and an intraclass correlation coefficient of 0.843 (*p* < 0.001), indicating a very high level of agreement between the reviewers. To enhance comprehensiveness and reduce selection bias, a consultation process with experts in medical education and educational technologies was conducted, in accordance with the optional consultation stage of the Arksey and O'Malley (2005) framework. This consultation involved discussions with professors of medical education and specialists in educational technologies to ensure that no relevant qualitative study or qualitative components of mixed‐methods studies had been overlooked. In addition, to collect grey literature, such as conference proceedings, a supplementary search was performed via Google Scholar. For conference abstracts identified through the supplementary Google Scholar search, if the full text was unavailable, the corresponding author was contacted by email (up to two attempts) to request the full text. Abstracts for which no response was received after two attempts were excluded from the review. All search results were imported into EndNote software.

This multi‐stage screening process, independent researchers with high inter‐rater agreement, and expert consultation ensured the precise, transparent, and unbiased selection of the included qualitative evidence and mixed‐methods components. The outcome of this stage was a selected set of original qualitative articles and the qualitative components of mixed‐methods studies that were used for thematic extraction and analysis in the subsequent stages of the scoping review. To promote transparency, the selection process was documented in a PRISMA flow diagram (Figure 1), which records the number of records at each stage from initial identification to the final inclusion of twelve studies.

### 2.4 Stage 4: Data Charting

To ensure the extraction of relevant and reliable data, a structured data extraction form was developed. To validate this form, four articles were randomly selected from the included studies. These articles were independently reviewed by two researchers, who extracted data to assess the form's consistency and alignment with the research question. Following discussion and refinement, the final form was organized under two main headings:

- *Study Characteristics:* Including authors, article title, year of publication, country of study, publication source, sample size, and target population.
- *Research Focus:* Encompassing the study's aim, qualitative methodology (type of qualitative method, data collection method, analysis approach), and key findings.

To enhance accuracy, any discrepancies in data extraction between researchers were resolved through group discussion or consultation with a third researcher. This process ensured that the data extraction form remained consistently aligned with the study's objectives in a systematic manner.

The extracted data were coded using a multi‐step approach inspired by qualitative content analysis:

*Open Coding:* Two researchers independently reviewed the articles, noting key concepts and themes, and generated initial codes to describe the core domains of each study. The qualitative analysis software *MAXQDA* was employed to manage and analyze the data, facilitating code organization, tracking relationships between categories, and providing preliminary data visualizations. Coding in *MAXQDA* was conducted collaboratively, with coding reports regularly reviewed among researchers to ensure coherence and precision.

*Category Development:* Codes were iteratively reviewed, and similar or related codes were merged and refined into conceptual categories. These categories were aligned with the research question and study objectives.

*Abstraction:* These categories were then synthesized at a higher level of abstraction into more overarching main themes to extract key findings in a more conceptual and structured manner.

### 2.5 Stage 5: Collating, Summarizing, and Reporting Results

In this stage, data extracted from original qualitative articles related to the role and significance of the metaverse in medical education were compiled, reviewed, analyzed, and reported. This process involved seven 90‐min sessions attended by the researchers and two experienced external experts (experienced Experienced faculty members in the fields of medical education and virtual reality technologies). The aim of Stage 5 was to provide a comprehensive mapping of existing findings in the scientific literature, identify key themes, research gaps, and challenges associated with the application of the metaverse in this field. Thematic analysis was conducted using the systematic six‐step approach proposed by Braun and Clarke (2006) [30], which includes:

1. Familiarization with Data (Text Interpretation): Extracted texts from the articles were thoroughly studied and interpreted to gain a comprehensive understanding of the qualitative content.
2. Generating Codes (Labeling Meaningful Text Segments): Meaningful sections of the texts were labeled using open coding, capturing initial concepts.
3. Identifying Themes (Determining Key Codes and Grouping Related Codes into Categories): Codes were grouped into preliminary categories based on conceptual similarities and relationships. These categories included themes such as simulation of realistic educational environments, enhanced motivation and engagement in learning, technical and infrastructural challenges, and others.
4. Reviewing Themes (Refining Categories and Establishing Relationships): Categories were revisited to ensure coherence and distinctiveness, with some codes merged or redefined as needed.
5. Defining and Naming Themes (Establishing Hierarchy and Significance): Each theme was assigned a clear label and precise definition to articulate its relevance to the research question.
6. Reporting Results (Organizing Findings into Meaningful Themes): The final themes were presented in a cohesive structure, illustrating the role and significance of the metaverse in medical education.

This structured approach ensured that the findings were systematically synthesized, offering a nuanced and comprehensive perspective on the metaverse's contributions to medical education while maintaining alignment with the study's objectives.

### 2.6 Rigor and Trustworthiness

The criteria proposed by Guba and Lincoln [31] serve as a foundational framework for evaluating the quality of qualitative research. These criteria encompass credibility, transferability, dependability, and confirmability. Below, these criteria are elucidated in the context of the present study, accompanied by the strategies employed to ensure research quality. A summary of these strategies is presented in Table 2.

**Table 2:** Strategies for Meeting the Four Guba and Lincoln Criteria.

Strategies	Four Guba and Lincoln criteria			
	Credibility	Transferability	Dependability	Confirmability
Triangulation	✓			
Peer debriefing	✓			
Iterative data review	✓			
Thick description		✓		
Comparison with related contexts		✓		
Audit trail			✓	
Use of standardized protocols			✓	
Team‐based review			✓	
Reflexivity				✓
External expert review				✓

(a) *Credibility:* Credibility pertains to ensuring the accuracy and validity of the research findings. To achieve this, the researchers implemented the following strategies:

- Triangulation: To enhance credibility, data were collected from multiple sources, including original qualitative articles and the qualitative components of mixed‐methods studies. This approach facilitated the validation of findings from diverse perspectives.
- Peer Debriefing: Extracted findings and themes were reviewed by independent researchers to verify the accuracy of data interpretation.
- Iterative Data Review: Data analysis was conducted iteratively, with continuous comparison across articles and themes to ensure the coherence and robustness of the findings.

(b) *Transferability:* Transferability refers to the potential for applying the study's findings to similar contexts or settings. The researchers employed the following strategies:

- Thick Description: The study provided a comprehensive description of the context (metaverse in medical education), methodology (qualitative scoping review), and characteristics of the included articles. This description included detailed accounts of main themes (e.g., technical and design challenges) and sub‐themes (e.g., the need for advanced hardware).
- Comparison with Related Contexts: Findings were contextualized by comparing them to related domains (e.g., surgical training, anatomy education), enabling readers to assess the applicability of results in other settings.

(c) *Dependability:* Dependability ensures that the research process is thoroughly documented and transparent, such that replication would yield comparable results. The researchers adopted the following strategies:

- Audit Trail: All research stages—from the search for articles in databases such as PubMed and Scopus to the selection of the final twelve articles, data coding, and theme extraction (e.g., regulatory and ethical frameworks)—were meticulously documented with transparency.
- Use of Standardized Protocols: The scoping review framework by Arksey and O'Malley (2005) was utilized to structure the research process. Additionally, the systematic six‐step approach by Braun and Clarke (2006) was applied for thematic analysis.
- Team‐Based Review**:** Theme analysis was independently conducted by multiple researchers to ensure consistency in coding and interpretation.

(d) *Confirmability:* Confirmability ensures that the findings are derived directly from the data and are not influenced by researcher bias. The researchers implemented the following strategies:

- Reflexivity: Potential biases (e.g., preconceptions about the metaverse) were examined and documented through reflective notes to minimize their impact on the analysis.
- External Expert Review: Themes and findings were evaluated by external experts to confirm that interpretations were consistent with the data.

These strategies collectively ensured that the study adhered to rigorous qualitative research standards, producing findings that are credible, transferable, dependable, and confirmable, while maintaining alignment with the research objectives.

## 3 Ethical Statement

This scoping review was conducted with full adherence to ethical principles in scientific research. All stages, from study design to reporting of results, were executed with integrity, transparency, and accountability. Data were presented without any distortion or manipulation. No conflicts of interest exist in this study, and all sources used have been appropriately cited. The findings have been reported with utmost accuracy and fidelity.

## 4 Results

### 4.1 Charting the Data

The initial searches, aligned with the objectives of this scoping review, yielded over 8,259 articles. Notably, although the researchers incorporated synonyms for “metaverse” to ensure no relevant studies were overlooked, this study specifically focuses on analyzing the role and significance of the metaverse in medical education, with an emphasis on original qualitative studies and the qualitative components of mixed‐methods studies. Unlike augmented reality (AR) and virtual reality (VR) technologies, the metaverse possesses unique characteristics, such as persistence and interactivity, which are not consistently present in AR and VR applications. Consequently, the researchers included only those articles that explicitly addressed the concept of the metaverse, ensuring a precise and valid response to the research question. After applying stringent inclusion criteria, only twelve articles met the eligibility requirements. Inter‐rater reliability was 0.836, with an intraclass correlation coefficient of 0.843 (*p* < 0.001). This limited number not only reflects the authors' rigorous and meticulous approach to source selection but also underscores a significant gap in the scientific literature concerning original qualitative studies on the metaverse in medical education. As Nagadeepa et al. (2024) similarly noted, there is a paucity of comprehensive studies on the metaverse in healthcare education [32]. Figure 1 was presented the PRISMA flow diagram for the study.

### 4.2 Characteristics of the Selected Studies

Data were extracted from the twelve included studies and charted according to the authors, study title, year of publication, country, publication source, sample size, and target population (Table 3). Additional information regarding the study objectives, study design, data collection methods, data analysis methods, and key themes or findings was also extracted and categorized in Table 4.

**Table 3:** Characteristics of included articles on the role of Metaverse in medical education.

No	Authors	Title of study	Publication year	Country	Publication source	Sample size	Target population
1	Lee et al. [33]	Exploring the potential use of the metaverse in nurse education through a mock trial	2023	South Korea	Nurse Education Today	14	Nursing students
2	Said [34]	Metaverse‐Based Learning Opportunities and Challenges: A Phenomenological Metaverse Human–Computer Interaction Study	2023	Egypt	Electronics	19	Metaverse and learning specialist
3	Turan Akdag et al. [35]	Transforming Medical Professional Training: Exploring the Metaverse in Education	2024	Germany	Pacific Asia Conference on Information Systems (PACIS)‐ PACIS 2024 Proceedings	4	Medical students
4	Mohammadzadeh et al. [36]	Principles of digital professionalism for the metaverse in healthcare	2024	Iran	BMC Medical Informatics and Decision Making	20	Medical information and health nformatics specialist
5	Irwin et al. [37]	The use of avatars: challenging longstanding approaches for experiential learning in nursing	2024	Australia	Interactive Learning Environments	24	Nursing student (11), instructor (8), nurse (5)
6	Ibi̇li̇ et al. [38]	Assessing the effectiveness and student perceptions of synchronous online flipped learning supported by a metaverse‐based platform in medical English education‐ A mixed‐methods study	2024	Türkiye	Education and Information Technologies	15	Medical students
7	Kim et al. [39]	Effects of metaverse‐based career mentoring for nursing students‐ a mixed methods study	2023	South Korea	BMC Nursing	51	Student (43) and nursing instructor (8)
8	Lee & Yoo [40]	Expectations and concerns about transitioning to face‐to‐face learning among Korean nursing students: A mixed methods study	2024	South Korea	PLOS ONE	14	Nursing students
9	Kim et al. [41]	Factors Associated with Continuous Use of a Cancer Education Metaverse Platform: Mixed Methods Study	2024	South Korea	Journal of Medical Internet Research	145	Radiology technicians, physicians, nurses
10	Nagadeepa et al. [32]	The “Metaverse Mania” in Healthcare Education: Students' Technology Acceptance	2024	India and Peru	Conference paper: Global Economic Revolutions: Big Data Governance and Business Analytics for Sustainability	214	Medical students and other healthcare students
11	Ma et al. [42]	The Research on The Application of Educational Metaverse in Experimental Teaching of Medical Physiology	2023	China	International Journal of Technology in Teaching and Learning	60	Medical students
12	Yoo et al. [43]	Effectiveness of Metaverse‐Based Collaborative Learning in Nursing Education: A Mixed‐Methods Study	2024	South Korea	Journal of Nursing Education	15	Nursing students

*Note:* The geographical distribution of the included articles across continents is presented in Figure 2.

**Table 4:** Data on objectives, Study design, Data collection method, Analysis method, and Key Findings from reviewed articles.

No	Authors	Objectives	Study design	Data collection method	Analysis method	Key findings
1	Lee et al. [33]	Exploration of nursing students' learning experiences utilizing mock trials within the metaverse platform (VRChat).	Qualitative approach	Reflective Journal	Thematic analysis	Key Features of the Metaverse Platform Core Characteristics of the Metaverse Platform Participation in Mock Trials within the VRChat Virtual Environment Metaverse‐Based Learning Utilizing Mock Trials
2	Said [34]	Investigation of the challenges and opportunities of metaverse technology with a human‐centered focus on learning, emphasizing human‐computer interaction (HCI).	Phenomenological approach	Unstructured in‐depth interviews, participant‐written essays, focus groups, and field notes	Thematic analysis	Opportunities: Hands‐On Training: The metaverse enables risk‐free simulations, such as surgical procedures or chemistry experiments, which are critical for medical education (p. 10). Game‐Based Learning: Incorporating gamification for laboratory training or medical simulations enhances engagement and learning outcomes (p. 10). Collaborative Knowledge Creation: The metaverse facilitates global collaboration among medical students and professionals, fostering shared knowledge development. Challenges: Immersive Design: There is a need for intuitive interfaces to prevent cognitive overload in medical education settings. Privacy and Security: Concerns arise regarding the protection of sensitive patient data used in medical simulations. Global Accessibility: The high cost of metaverse equipment may restrict access for medical students in less‐developed regions. Physical and Mental Health: Prolonged use of VR headsets may lead to fatigue or social isolation, potentially affecting students' well‐being. Governance: Robust regulations are required to manage the use of the metaverse in medical education, particularly concerning data management and ethical conduct.
3	Turan Akdag et al. [35]	Identification and characterization of opportunities and challenges associated with the use of the metaverse in medical education, achieved through a synthesis of scenario‐based qualitative interviews with experts and a literature review to gain a comprehensive understanding of effective metaverse applications in this field.	Qualitative approach	Semi‐structured interviews	Analyzing interview data using software f4analyse	The metaverse is well‐suited for both theoretical and practical education, particularly through the use of three‐dimensional models that enhance understanding of bodily structures. However, it is currently viewed as a complement to traditional education rather than a replacement. Acceptance of the metaverse is high, yet limitations in human interaction, such as the inability to convey facial expressions, position it as a supplementary tool to traditional education. Improving avatar representation could further increase its acceptance. The metaverse holds significant potential for safely simulating complex scenarios and improving access to medical education in remote areas. Nevertheless, it is presently utilized as an adjunct to traditional educational methods. Key challenges include technical limitations (e.g., realism of simulations and user‐friendliness), ethical concerns (e.g., reduced empathy due to limited human interaction), and accessibility issues. Privacy concerns were less prominent in the interviews.
4	Mohammadzadeh et al. [36]	Examination of the concept of digital professionalism and its relevance in the context of healthcare within the metaverse, guided by a set of principles for education, learning, and practice in healthcare professions.	Qualitative approach	Semi‐structured interviews	Thematic Analysis	The study identified ten core principles of digital professionalism for the metaverse in healthcare: privacy and security, informed consent, trust and integrity, accessibility and inclusivity, professional boundaries, evidence‐based practice, continuous education and training, collaboration and interoperability, feedback and improvement, and regulatory compliance.
5	Irwin et al. [37]	Evaluation of the effectiveness of using the Second Life metaverse platform for training and practicing clinical nursing skills in an undergraduate nursing program, alongside an assessment of its acceptance as an educational strategy by key stakeholders (students, instructors, and registered nurses).	Qualitative approach	Semi‐structured interviews	Thematic Analysis	Realism: All participant groups (students, instructors, and nurses) noted similarities between learning in Second Life and real‐world clinical environments. The platform was perceived as challenging yet manageable and safe. Bridging the Theory‐Practice Gap: Second Life was recognized as a valuable tool for addressing the theory‐practice gap, particularly in fostering higher‐level skills such as communication, prioritization, and clinical decision‐making. Limitations: Certain practical skills (e.g., specific technical competencies) could not be effectively taught in Second Life, though soft skills and clinical reasoning were well‐developed in this environment. Curriculum Recommendations: All groups advocated for broader integration of Second Life into nursing curricula, recommending a structured and scaffolded approach to incorporating this technology.
6	Ibi̇li̇ et al. [38]	Qualitative Objective: Investigation of students' opinions and perspectives regarding the educational efficacy of synchronous online learning based on the flipped classroom model, supported by the metaverse platform (Spatial AR), in teaching medical English.	Mixed‐Method Approach	Semi‐structured interviews	Thematic Analysis in qualitative part	Qualitative Findings: The findings highlighted the positive impact of the metaverse‐supported flipped classroom approach on motivation, sustained learning, and language skills, particularly reading. However, challenges such as technical difficulties and the time‐intensive nature of activities were also noted.
7	Kim et al. [39]	Qualitative Objective: Exploration of the experiences of nursing instructors and students with metaverse‐based career guidance.	Mixed‐Method Approach	Focus group interviews	Thematic Analysis in qualitative part	Qualitative Findings: Three primary themes were derived from the focus group interviews: 1. Unconstrained Candid Interviews: ◦ Avatar‐based anonymity alleviated psychological pressure. ◦ Trainees were able to freely pose sensitive questions. ◦ Instructors could provide more forthright and honest responses. 2. Satisfaction with Authentic Dialogues and Platform Functionality: ◦ Participants expressed contentment with acquiring new and precise information relevant to the research topic. ◦ The use of realistic avatars and simulated environments enhanced focus and a sense of realism. ◦ The metaverse platform was appreciated as a novel and engaging method for guidance, offering interaction via avatars and freedom from spatial‐temporal constraints. 3. Expectations for an Even More Optimized Program: ◦ Limitations reported included the inability to customize avatars, limited variety in movements and facial expressions, and the time‐intensive nature of program installation. ◦ Participants sought features to foster greater intimacy, such as improved interactions and the ability to gauge others' reactions, as the lack of identity cues and interaction constraints hindered the development of rapport.
8	Lee & Yoo [40]	Qualitative Objective: Assessment of nursing students' satisfaction with learning and their anxiety concerning the complete transition to in‐person learning post‐COVID‐19, focusing on their learning methods, expectations, and concerns.	Mixed‐Method Approach	Semi‐structured interviews	Inductive Content Analysis	Qualitative Findings: Three categories were identified: (1) the burden of unfamiliar and unpredictable learning conditions, (2) considerations for in‐person learning to enhance satisfaction, and (3) a turning point that compensates for feelings of deprivation in university life. Students expressed concerns about unexpected changes, unfamiliar relationships, and the effectiveness of learning, yet they anticipated more dynamic interactions and fairer assessments.
9	Kim et al. [41]	Qualitative Objective: Examination of participants' feedback on the user experience of a metaverse educational center sub‐platform and the factors influencing their intent to continue using it.	Mixed‐Method Approach	Collection of qualitative data through open‐ended questions included at the end of the quantitative section of a questionnaire	Reflexive Thematic Analysis ‐ Content analysis approach	Qualitative Findings: From 276 feedback statements, 21 described advantages (e.g., usefulness, immersion, satisfaction), while 255 highlighted areas for improvement (e.g., limited interactivity in content, lack of content variety, device weight, and low image quality).
10	Nagadeepa et al. [32]	Qualitative Objective: Understanding students' perspectives on the metaverse in healthcare education, exploring their stage of metaverse adoption, and identifying challenges they may encounter when using metaverse virtual reality environments.	Mixed‐Method Approach	Extraction of qualitative data from open‐ended response questions in the quantitative section of a questionnaire	Inductive analysis	Perceived Challenges by Students: Health concerns associated with metaverse use, particularly eye‐related issues. Distractions within the metaverse learning environment. Difficulties in maintaining discipline and focus during virtual classes. Lack of technological knowledge and skills. Adverse effects on social interactions and social life. Resistance to technology dependence. Risks related to privacy and cybercrimes.
11	Ma et al. [42]	Qualitative Objective: Investigation of the potential to enhance medical physiology education through the use of a metaverse‐based experimental training system.	Mixed‐Method Approach	Derivation of qualitative data from open‐ended response questions within the quantitative component of a questionnaire	Inductive analysis	Qualitative Findings: High satisfaction with the virtual experimental training model in the metaverse. Enhanced intuition and clarity in understanding experimental processes. Strong proficiency in mastering motor‐respiratory regulation skills for the experimental subject. Increased enthusiasm and motivation for learning experimental skills.
12	Yoo et al. [43]	Qualitative Objective: Exploration of the effects of using the metaverse virtual space for collaborative learning among nursing students	Mixed‐Method Approach	Focus Group Interviews & Semi‐structured interviews	Content Analysis	Qualitative Analysis: The qualitative analysis identified three primary categories: Unfavorable changes in the learning environment. Discovery of the learning environment's potential to foster engagement. Recommendations for developing the metaverse learning environment. Students described the metaverse as an entertaining, engaging, and effective environment for collaborative learning. However, they expressed concerns about unfamiliarity and apprehension regarding changes to learning methods. Suggestions for improving the metaverse learning environment included developing diverse functionalities, refining teaching methods, and ensuring network stability.

### 4.3 Distribution by Year of Publication

Regarding the year of publication, four of the twelve included studies (33.3%) were published in 2023, whereas eight studies (66.7%) were published in 2024. Thus, the majority of the included studies were published in 2024.

### 4.4 Geographical Distribution

**Figure 2:**
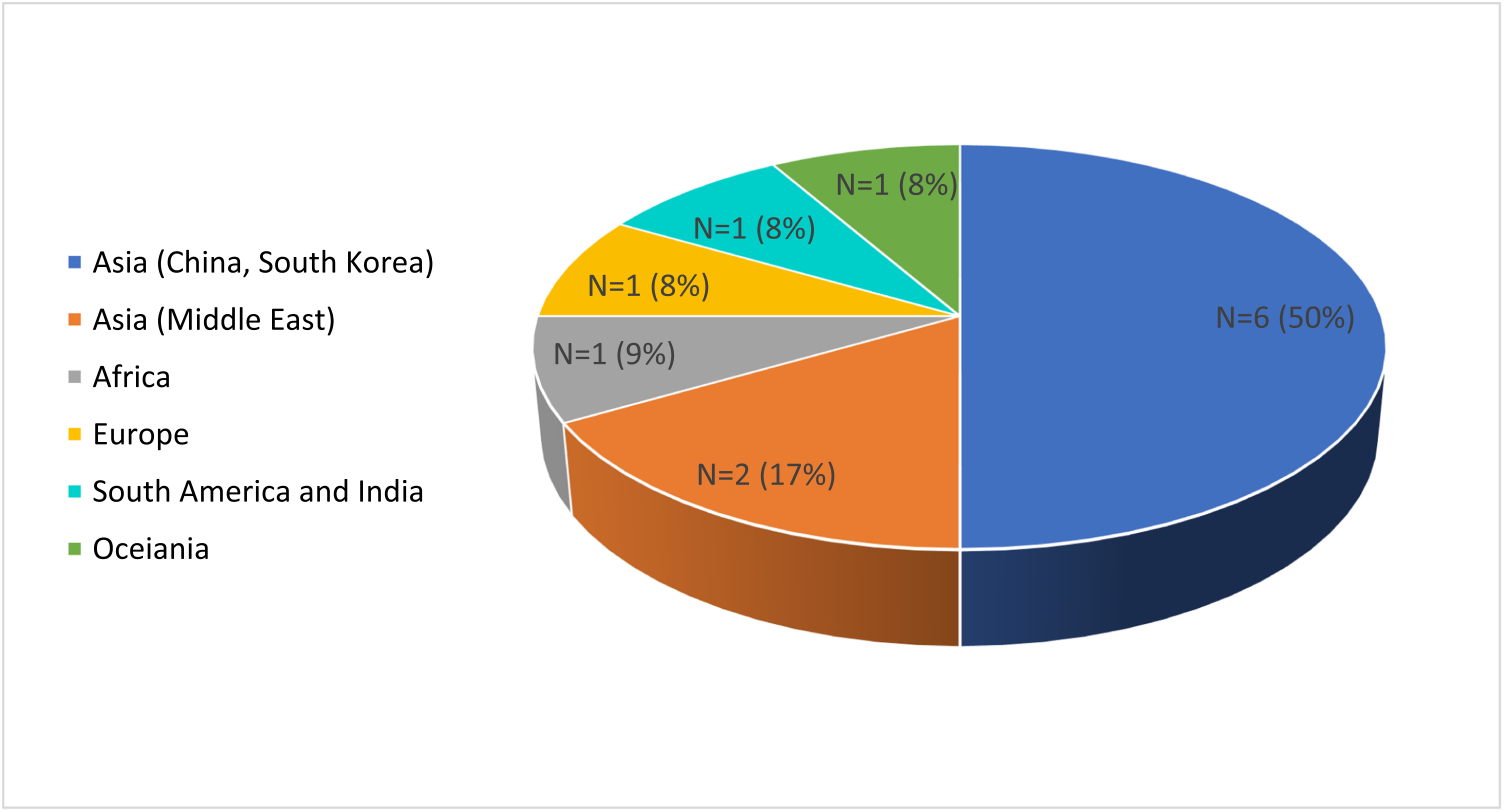
Geographical distribution of the included studies by continent.

Regarding geographical distribution, Asia was the most represented continent, accounting for eight of the twelve included studies (66.7%). These studies were conducted in South Korea [33, 39, 40, 41, 43], Iran [36], Türkiye [38], and China [42]. One study was conducted in Africa (Egypt) [34], one in Europe (Germany) [35], and one in Oceania (Australia) [37]. In addition, one study involved participants from India and Peru, representing a cross‐continental contribution from Asia and South America [32]. The total number of participants across the twelve studies included in this review was 595 (Table 3; Figure 2).

### 4.5 Methodological Characteristics of the Included Studies

Of the twelve studies reviewed, six employed a qualitative approach, while the remaining six used a mixed‐methods approach. For the mixed‐methods studies, only the qualitative components were considered in the present review to align with the study objectives.

Regarding data collection methods, one study used unstructured in‐depth interviews (34), one used reflective journal [33], five employed semi‐structured interviews (35, 36, 37, 38, 40), two used focus group interviews [39, 43], and three collected qualitative data through open‐ended questions included at the end of quantitative questionnaires [32, 41, 42] (Table 4).

### 4.6 Analysis of Key Themes in the Final Studies

The analysis of key themes in the final studies revealed eight primary themes: immersion and sense of reality in the metaverse, educational benefits of the metaverse, technical and design challenges, privacy and security concerns, social and psychological impacts, accessibility and inclusivity, regulatory and ethical frameworks, and educational innovations and teaching methodologies. The themes and sub‐themes derived from the analysis of the reviewed studies are presented in Table 5.

**Table 5:** Fundamental issues related to the role of the Metaverse in e‐learning in the field of medical education.

No	Main theme	Sub‐themes
1	Immersion and sense of reality in the metaverse	Realism and Similarity to the Real World Immersive and Dynamic Interaction Simulation of Realistic Educational Environments
2	Educational benefits of the metaverse	Enhancement of Skills and Learning Increased Motivation and Learning Engagement Safe and Cost‐Effective Learning Flexibility and Autonomy in Learning
3	Technical and design challenges	Design Complexities Technical and Infrastructural Challenges User Experience and Usability Difficulties
4	Privacy and security concerns	Privacy and Data Security Data Management and Responsibility Contradictions and Privacy Challenges
5	Social and psychological impacts	Psychological Impacts Physical Impacts Social Inequities and Gaps
6	Accessibility and inclusivity	Technological Access Barriers Design for Inclusivity and Universal Access
7	Regulatory and ethical frameworks	Need for Governance and Regulations Legal and Transnational Challenges Accountability and Professional Conduct
8	Educational innovations and teaching methodologies	Innovative Teaching Methodologies Transformation of Educational Curricula Collaborative and Global Learning

### 4.7 Thematic Synthesis of Findings

Thematic analysis of the 12 included studies revealed eight main themes. The number of studies associated with each of the eight main themes is as follows: the distribution of evidence across the themes was uneven. The theme Immersion and Sense of Reality in the Metaverse was reported in 8 studies [33, 34, 35, 37, 39, 41, 42, 43], Technical and Design Challenges in nine studies [32, 33, 34, 35, 37, 38, 39, 41, 43], Educational Innovations and Teaching Methodologies in 8 studies [33, 35, 36, 37, 38, 39, 42, 43], Educational Benefits of the Metaverse in 10 studies [33, 34, 35, 36, 37, 38, 39, 41, 42, 43], Social and Psychological Impacts in 7 studies [32, 34, 35, 37, 39, 41, 42, 43], Accessibility and Inclusivity in 5 studies [32, 34, 35, 36, 37], Privacy and Security Concerns in 5 studies [32, 34, 35, 36, 39], and Regulatory and Ethical Frameworks in 3 studies [34, 35, 36]. This distribution indicates that stakeholders currently emphasize experiential and educational gains while simultaneously identifying persistent technical barriers; regulatory and ethical frameworks, privacy and security concerns, ethical issues, and accessibility and inclusivity remain relatively less examined in the qualitative literature.

*Immersion and Sense of Reality in the Metaverse:* Eight studies highlighted immersion as a pivotal and distinguishing feature of the metaverse relative to conventional e‐learning. Participants described a heightened sense of presence and reality when interacting through avatars in persistent three‐dimensional environments [33, 34, 35, 37, 39, 41, 42, 43]. Lee et al. reported that nursing students in the VRChat environment found the virtual courtroom sufficiently realistic to support authentic role‐playing [33]. Erwin et al. observed that students, instructors, and clinical nurses evaluated Second Life scenarios as comparable to real clinical settings [37]. Kim et al. [39, 41] and Ma et al. [42] likewise emphasized that realistic avatars and simulated laboratories enhanced concentration and intuitive understanding of procedures. Nevertheless, the quality of immersion was strongly platform‐dependent; more mature environments (such as VRChat and Second Life) generated stronger presence, whereas custom platforms were constrained by limitations in avatar personalization, facial expressions, and image quality.

*Educational Benefits of the Metaverse:* Ten studies documented the perceived educational benefits. These benefits centered on four main axes: skill enhancement, increased motivation and engagement, the opportunity for safe and cost‐effective practice, and temporal‐spatial flexibility. The safe simulation of high‐risk or rare scenarios was the most frequently reported benefit [33, 34, 35, 37, 42]. Students were able to practice clinical and laboratory procedures without risk to the patient. Motivation and engagement were also widely reported; participants described the environment as novel, entertaining, and engaging, and an increase in enthusiasm for specialized content such as medical terminology and physiology was observed [38, 41, 42, 43]. Avatar‐based anonymity in mentoring sessions reduced psychological pressure and facilitated the raising of sensitive questions [39]. Overall, stakeholders regarded the metaverse as a powerful complement to traditional education rather than a complete substitute for it [35, 37].

*Technical and Design Challenges:* Nine studies identified technical and design challenges as the most important barrier to effective use. Recurring issues included the need for advanced hardware and stable high‐speed internet, a complex installation process, the weight and discomfort of the device, low image quality, limited content interactivity, and a non‐intuitive user interface [32, 34, 35, 37, 38, 39, 41, 43]. Limitations in avatar personalization and the range of movements also reduced realism and sustained engagement [39, 41]. Ready‐made platforms such as VRChat, Second Life and Spatial had faster deployment but offered less educational flexibility [33, 37, 38], whereas custom‐developed educational platforms demonstrated greater alignment with curriculum objectives but suffered from greater technical immaturity [39, 41, 42, 43]. Network instability was also reported as a practical barrier in collaborative learning [43].

*Privacy and Security Concerns:* Five studies addressed privacy and data security concerns [32, 34, 35, 36, 39]. Stakeholders expressed concerns regarding the protection of sensitive patient data in simulations [34], the risks of cybercrime [32], and the lack of clear data governance protocols [34, 36]. Mohammadzadeh et al. regarded privacy, security, and informed consent as fundamental principles of digital professionalism in the healthcare metaverse [36]. In contrast, these concerns were less prominent in the study by Turan Akdag et al. [35]. Avatar anonymity was raised both as an educational advantage and as an issue of identity privacy [39].

*Social and Psychological Impacts:* Seven studies examined the social and psychological consequences [32, 34, 35, 37, 39, 40, 43]. Positive effects included a reduction in social anxiety through avatar anonymity, the possibility of more candid dialogue in mentoring sessions, and the creation of a sense of global connectedness [39]. In contrast, negative effects reported included ocular and physical fatigue resulting from prolonged headset use, distraction, difficulty in maintaining concentration and discipline, a reduction in non‐verbal cues and consequently diminished empathy, and the potential for social isolation [32, 34, 35, 43]. Lee and Yu [40] described the ambivalence of students upon returning to face‐to‐face learning after the virtual experience, which manifested as a combination of relief and a sense of deprivation.

*Accessibility and Inclusivity:* Five studies raised issues of access and inclusion [32, 34, 35, 36, 37]. High equipment costs and infrastructural requirements were identified as a factor excluding students in resource‐limited regions. At the same time, the metaverse was considered a potential tool for enhancing access among learners in remote areas or those with physical limitations, provided that inclusive design principles are observed [35, 36, 37]. Mohammadzadeh et al. [36] explicitly included access and inclusion among the principles of digital professionalism. Nevertheless, current implementations still fall considerably short of universal design.

*Regulatory and Ethical Frameworks:* Three studies fundamentally addressed regulatory and ethical frameworks [34, 35, 36]. Participants and experts called for clear regulations in the domains of data management, professional boundaries, validation of simulations, and transnational legal issues [45]. Mohammadzadeh et al. presented the most comprehensive set of principles, encompassing regulatory compliance, professional boundaries, informed consent, and professional integrity [36]. The relative scarcity of evidence on this theme indicates a significant research and policy gap; technical and educational development has outpaced the formation of ethical and legal safeguards.

*Educational Innovations and Teaching Methodologies:* Eight studies described the metaverse as a catalyst for educational innovation [33, 35, 36, 37, 38, 39, 42, 43]. The reported innovations included mock trial‐based education, a synchronous flipped classroom supported by the Spatial platform, avatar‐based career mentoring, collaborative learning in persistent virtual spaces, and experimental physiology laboratories [33, 35, 36, 37, 38, 39, 42, 43]. Stakeholders recommended structured, curriculum‐level integration rather than isolated additive activities and called for richer functionalities, more diverse content, and teaching methods aligned with the affordances of persistent virtual worlds [37, 39, 43]. A shared observation was that the metaverse holds greatest value when it enables interactions that are difficult or impossible in conventional classrooms (such as anonymous sensitive questioning, simultaneous global collaboration, and risk‐free practice of rare events) [10].

Overall, the findings extracted from 12 studies indicate that stakeholders placed the greatest emphasis on immersion, motivation, engagement, and the opportunity for safe practice of skills. These themes were reiterated across a larger number of studies. In contrast, themes related to regulatory and ethical frameworks, privacy and security concerns, and access and inclusivity received attention in fewer studies. Clear differences were also observed among the various platforms: higher‐fidelity and established environments (such as VRChat and Second Life) were more frequently associated with reports of a sense of reality and satisfaction, whereas targeted and customized educational platforms were more often reported alongside technical challenges and design limitations. The distribution of evidence across the themes was unbalanced, and the primary focus of the studies rested on short‐term experiential and educational aspects.

**Figure 3:**
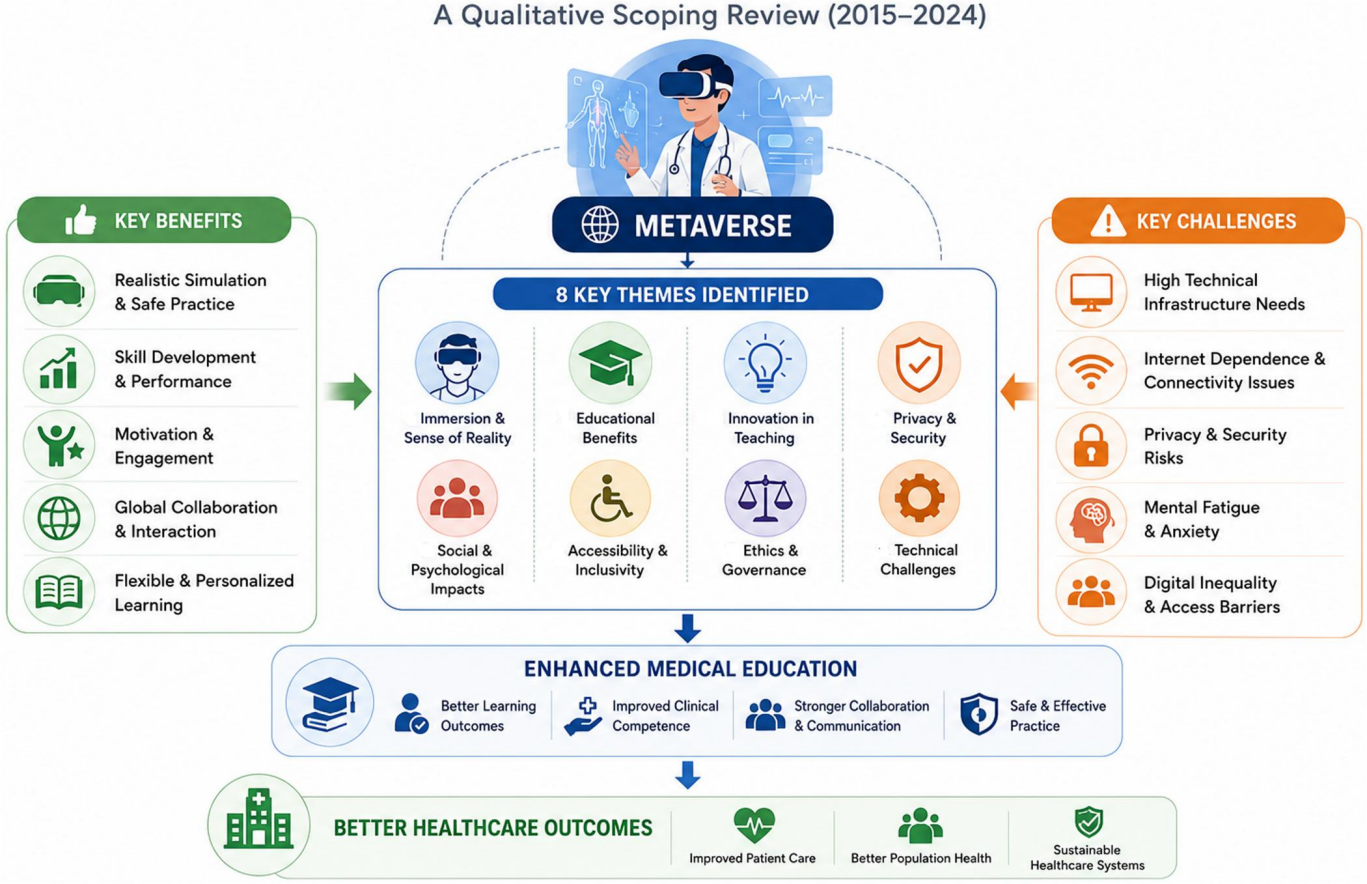
Thematic framework of the role of the Metaverse in electronic learning in medical education.

A schematic summary of the main themes and sub‐themes identified in the scoping review is presented in Figure 3.

## 5 Discussion

The aim of this qualitative scoping review was to identify, analyze, and synthesize qualitative evidence regarding stakeholders' perspectives and experiences concerning the application, role, and significance of the metaverse in medical education. Thematic analysis of the twelve final studies revealed eight principal themes. Immersion and perceived educational benefit were the most frequently reported, while technical and design limitations were the most frequently cited barriers. Privacy, access, psychosocial effects, and ethical–regulatory arrangements have attracted relatively less qualitative attention, indicating that experiential and educational claims have outpaced the development of safeguards and justice‐oriented design. The narrative below interprets each theme in relation to the available evidence and the broader international literature and then synthesizes the themes.

Immersion and sense of reality in the metaverse was one of the features that was consistently reported in the included studies. Stakeholders evaluating Second Life described the clinical scenarios as comparable to the real environment, while remaining challenging, manageable, and safe [37]. These findings are consistent with conceptual descriptions of the metaverse as a persistent and interactive three‐dimensional space that can enhance the sense of presence beyond conventional e‐learning [6, 15, 44]. The maturity of the platform mattered. Established environments such as VRChat and Second Life were more often associated with a strong sense of presence, whereas custom educational platforms frequently faced limitations in avatar customization, range of movement, and image quality [39, 41, 43]. High‐fidelity simulations in anatomy and surgery can enhance spatial understanding, provided that hardware and content quality are adequate. Institutional examples of the use of virtual reality and mixed reality for teaching anatomy and neurosurgery (such as patient‐model‐based neuroanatomy courses) demonstrate this potential; however, their educational value depends more on instructional design than on the technology itself [20, 44]. Risk‐free practical training for the patient, which was repeatedly emphasized by participants in the included studies [33, 34, 35, 37, 42, 45], is consistent with simulation research indicating the role of risk‐free practice in experiential learning and in increasing learner self‐confidence [20, 45].

The reported educational benefits of the metaverse, according to the findings of ten of the twelve studies, revolved around four main themes: enhancement of skills and learning, increased motivation and engagement, opportunities for safe and relatively low‐cost practice, and temporal–spatial flexibility [33, 34, 35, 36, 37, 38, 39, 41, 42, 43]. These stakeholder perspectives are consistent with broader evidence indicating that immersive and metaverse‐related tools can support motivation, interaction, and access to rare clinical cases [45, 48, 49]. Platforms designed for procedural practice (such as virtual‐reality surgical simulators) and holographic anatomy systems have, in separate studies, been associated with increased engagement and, in some procedures, with improvements in technical performance indicators. These platforms enable students to refine surgical and anatomical skills in simulated environments and to cultivate interactive, dynamic learning experiences that closely mirror actual clinical practice [20, 44]. Petrigna et al. (2022) demonstrated that the metaverse furnishes a quasi‐realistic environment in which students can engage in critical thinking, manipulate scenarios that are impossible in real life, and develop a strong desire to learn [46]. Balcıoğlu et al. (2023) likewise noted that the metaverse strengthens learners' positive disposition toward acquiring diverse learning experiences [47]. The metaverse confers multiple educational advantages, including interactive and personalized programs, immediate feedback, opportunities for both independent and collaborative learning, and enhanced student self‐direction [48]. Evidence from knee arthroplasty training indicates that virtual‐reality simulation can improve completion of surgical steps, efficiency, and certain performance indicators among trainees, although results vary according to the task, the comparator, and the outcome measure. The technology also fosters global collaboration through virtual platforms [45, 49].

Technical and design challenges were the most frequently reported barrier (nine studies). The advancement of medical education is highly dependent on the expansion of technology [50]. Recurring problems included the need for advanced hardware and stable high‐speed internet, a complex installation process, headset weight and discomfort, low image quality, limited content interaction, and a non‐intuitive user interface [32, 34, 35, 37, 38, 39, 41, 43]. These findings are consistent with feasibility studies showing that many medical students have little prior experience with immersive environments. In a metaverse‐based anatomy education study, 63.3% of participating sixth‐year medical students had no previous experience with immersive virtual environments, and almost all were not regular users of this technology [51]. When interfaces are not adapted to this learner profile, cognitive load is transferred from clinical content to device management [51]. Therefore, designs that simplify interaction, reduce setup burden, and align platform capabilities with curriculum objectives are not peripheral technical details, but a condition of educational usefulness [15, 22, 51].

Data security in the metaverse demands particular attention, as stakeholder trust is pivotal for its adoption [20, 52]. Privacy and data security concerns, particularly regarding patient data, are paramount in the metaverse. Robust protective frameworks are essential to manage data and prevent privacy breaches, with the lack of global standards exacerbating this challenge [15, 22, 44]. Privacy and security were addressed in five of the included studies. Data security in the metaverse requires particular attention, as stakeholder trust is essential for its adoption [20, 52]. In contrast, these concerns were less prominent in one expert interview study [35]. These concerns have also been reflected in broader analyses of metaverse applications in medicine, which indicate that confidentiality mechanisms and data protection standards remain underdeveloped relative to the sensitivity of health information [52, 53, 54]. Yang et. al emphasized the importance of addressing privacy and security concerns in metaverse applications and noted that emerging technologies often give rise to challenges such as data security issues. While the metaverse holds promise, its mechanisms for ensuring confidentiality and data protection remain inadequate [53]. It is crucial to recognize that the metaverse, as a virtual space, may not fully adhere to the required standards for information security and privacy [54]. Privacy and data security concerns, particularly regarding patient data, are of considerable importance in the metaverse. Robust protective frameworks for data management and the prevention of privacy breaches are essential, and the lack of global standards exacerbates this challenge [15, 22, 44]. The qualitative literature reviewed in this review remains limited on this theme; stakeholder concerns are evident, yet detailed reports of actual data flows, consent practices, and institutional measures are scarce.

According to studies, the Metaverse has dual social and psychological impacts that are both positive and negative. In one study, virtual reality‐based exposure therapy (VRET) for social anxiety proved more effective than traditional methods, increasing patient satisfaction [20]. However, challenges such as reduced personal connection and distractions during virtual sessions were noted. The metaverse can improve mental health and collaboration but requires careful management. These impacts influence students' learning experiences and mental well‐being [48, 49]. The metaverse boosts motivation, which in turn enhances education and learning. By creating an active state in learners through realistic yet virtual conditions, it allows individuals to test acquired skills in simulated environments [55]. Augustine et al. (2022) found that incorporating gamification within the metaverse positively influences learners' interest, motivation, and comprehension [56]. Similarly, Lopez et al. (2022) highlighted that the metaverse experience not only improves learning outcomes but also increases student motivation [57]. Conversely, prolonged use of the metaverse may induce psychological effects such as fatigue or anxiety. Access disparities could exacerbate social inequities, necessitating further research to understand these impacts [15, 22, 44]. Thus, the included educational studies present a dual picture: the same immersive features that increase engagement can produce fatigue, reduced richness of interpersonal relationships, and attentional strain, which necessitates further comparative studies.

Although the metaverse has not yet reached a stable state in education, its application is rapidly expanding [8]. It makes medical education more accessible for students in remote areas or with disabilities [58]. Virtual consultations and telerehabilitation improve access, but equipment costs and technical issues pose barriers. Thoughtful learning design for students with visual or auditory impairments is essential. The metaverse can reduce educational disparities but requires investment. Inclusivity is critical for educational equity [20, 45]. The metaverse must be accessible to individuals with physical limitations, as technological barriers such as high costs and infrastructural requirements can limit inclusion. Inclusive designs are vital for global access [15, 22, 32, 34, 45].

Only three of the included studies substantively addressed regulatory and ethical frameworks. Integration of the metaverse into medical education requires robust regulatory and ethical frameworks. Transparency and accountability are crucial for maintaining trust and integrity in virtual environments [59]. Clear standards for safety and efficacy are essential, as the absence of standardized frameworks could hinder metaverse adoption. These frameworks ensure trust and quality [48, 52]. Regulations to uphold ethical standards in the metaverse are vital, with transnational legal challenges and professional accountability requiring attention [15, 22, 44]. The limited volume of qualitative evidence in this theme is itself a finding: technical and educational development has outpaced the formation of ethical and legal measures [45, 52, 59]. Transparency, simulation validation and professional behaviour in persistent virtual environments have not yet been sufficiently specified for the education of high‐risk health professions.

The metaverse facilitates innovations such as AI‐driven adaptive learning, interdisciplinary training, and access to rare medical cases [45]. It enhances collaboration and teamwork skills, revolutionizing teaching methods and promoting personalized learning. These innovations can modernize medical education [49, 58]. As a form of virtual reality, the metaverse serves as an alternative to traditional virtual environments [60] and can be integrated alongside the real world to facilitate learning [10]. It enables innovative teaching methods, such as global collaborative learning, while transforming curricula through interactive simulations [15, 22, 44]. Chen et al. (2022) reported that metaverse use not only enhances student learning outcomes but also increases their willingness to adopt this technology [61].

Overall, stakeholders currently emphasise immersion, motivation, engagement and safe skills practice. Technical barriers have been well documented. Privacy, access, psychosocial harm and corporate governance have been acknowledged but remain less examined in qualitative research. Platform differences are consistent: higher‐fidelity and more mature environments have often been associated with reports of presence and satisfaction, whereas purpose‐built educational platforms have often been associated with reports of technical immaturity. The evidence continues to centre on short‐term experiential accounts. Therefore, claims concerning sustained learning, clinical transfer and population‐level equity require a more balanced programme of qualitative, mixed‐methods and longitudinal research. All of the above interpretations should be studied in light of the scope and limitations of the study itself, which are described below.

### 5.1 Strengths, Limitations and Recommendations for Future Research

The main strengths of this study include the following: a rigorous design as a qualitative scoping review based on the six‐stage framework of Arksey and O'Malley, a comprehensive and systematic search of English and Persian databases accompanied by the application of PRISMA‐ScR, very high inter‐rater agreement (Cohen's kappa = 0.836 and ICC = 0.843), the thematic extraction and analysis of eight key themes from twelve qualitative studies and the qualitative sections of mixed‐methods studies (Tables 3, 4, 5), and the assurance of rigor based on the criteria of Guba and Lincoln, which collectively led to the identification of research gaps and the presentation of a comprehensive map of stakeholders' perspectives on the role of the metaverse in medical education.

A primary limitation of this study was the scarcity of original qualitative studies available for comprehensive analysis. Nevertheless, the most relevant studies were meticulously selected for evaluation. As an emerging technology, the metaverse has primarily been explored through theoretical reviews or quantitative studies. The paucity of original qualitative research on the metaverse in medical education stems from the technology's novelty, technical complexity, and the predominance of quantitative approaches. To address this, qualitative methodologies tailored to virtual environments, particularly the metaverse, should be developed, financial and technical support should be increased, and interdisciplinary collaborations should be strengthened. Such measures could facilitate a deeper understanding of the metaverse's role in medical education.

The following recommendations provide guidance for future research to fully realize the potential of the metaverse in medical education:

- Conduct qualitative studies to explore students' experiences with the application of the metaverse in medical education.
- Undertake qualitative and participatory studies involving policymakers, ethicists, and educational stakeholders to develop regulatory and ethical frameworks that ensure data security, privacy, and educational standards.
- Pursue interdisciplinary research to establish global standards for privacy and ethics in the metaverse [15].
- Conduct in‐depth studies to understand the long‐term effects of the metaverse on mental health, such as fatigue or anxiety [22].
- Perform longitudinal research to evaluate the impact of the metaverse on learning outcomes and clinical performance of medical students [44].

## 6 Conclusion

The evidence from this qualitative review of stakeholders indicates that the metaverse is currently valued in medical education primarily as an immersive complement to conventional instruction: it can support presence, motivation, and the safe rehearsal of uncommon or high‐risk tasks. This capacity is not uniform. Educational gain is closely tied to platform maturity and instructional design, while technical burden, incomplete privacy governance, unequal access, and weakly specified ethics still constrain responsible use. The qualitative literature maps these tensions more clearly than it demonstrates lasting effects on competence or care.

In education and clinical practice, the implication is selective adoption rather than wholesale replacement. Metaverse environments make possible what wards and classrooms cannot readily offer—risk‐free procedural practice, anonymous mentoring, and distributed collaboration—and are weakest where tactile skill, empathy, and in‐person clinical assessment are central to the work. The metaverse is therefore best treated as a complementary curricular scaffold, not a substitute for supervised clinical work.

In policy, infrastructure and standards must precede scale. Institutions and regulators need minimum expectations for data protection, simulation validity, professional conduct in persistent virtual spaces, and equitable access; technical challenges, privacy concerns, access issues, and the need for robust regulatory frameworks must be addressed. Without these, technological expansion may widen inequality and erode trust.

In research, the present imbalance of evidence sets the agenda: most available evidence is confined to short‐term learner experience, and little is yet known about how the technology alters durable learning, long‐term mental health, or the implementation of educational policy. The next step is not to re‐describe the novelty of the metaverse, but to design studies that show whether the advantage of immersion persists or dissipates once it enters the curriculum, the clinical environment, and conditions of unequal resources.

## Author Contributions

**Fariba Khanipoor:** conceptualization, investigation, writing – original draft, methodology, writing – review and editing, formal analysis, validation, data curation, visualization. **Zahra Karimian:** conceptualization, investigation, methodology, validation, visualization, writing – review and editing, project administration, supervision.

## Conflicts of Interest

The authors declare no conflicts of interest.

## Declaration of Artificial Intelligence Use

We declare that **Grok AI** was used solely to translate the Persian manuscript text into English and to improve grammatical accuracy, clarity, sentence structure, word choice, and consistency in tone and style. **ChatGPT (OpenAI)** was used solely to assist with the graphical design and visualization of two figures. All manuscript content, intellectual contributions, scientific interpretations, analyses, and conclusions were developed and verified by the authors, who take full responsibility for the accuracy and integrity of the work.

## Transparency Statement

The lead/Corresponding author (Zahra Karimian) affirms that this manuscript is an honest, accurate, and transparent account of the study being reported; that no important aspects of the study have been omitted; and that any discrepancies from the study as planned (and, if relevant, registered) have been explained.

## Data Availability

The data analyzed during the current study are available from the corresponding author on reasonable request.
